# Orexin 1 receptor antagonists in compulsive behavior and anxiety: possible therapeutic use

**DOI:** 10.3389/fnins.2014.00026

**Published:** 2014-02-13

**Authors:** Emilio Merlo Pich, Sergio Melotto

**Affiliations:** ^1^Neuroscience DTA, F. Hoffman – La RocheBasel, Switzerland; ^2^Legnago, Italy

**Keywords:** drug addiction, relapse, binge eating, repetitive behavior, emotion, OX1 receptor antagonist, GSK1059865

## Abstract

Fifteen years after the discovery of hypocretin/orexin a large body of evidence has been collected supporting its critical role in the modulation of several regulatory physiological functions. While reduced levels of hypocretin/orexin were initially associated with narcolepsy, increased levels have been linked in recent years to pathological states of hypervigilance and, in particular, to insomnia. The filing to FDA of the dual-activity orexin receptor antagonist (DORA) suvorexant for the indication of insomnia further corroborates the robustness of such evidences. However, as excessive vigilance is also typical of anxiety and panic episodes, as well as of abstinence and craving in substance misuse disorders. In this review we briefly discuss the evidence supporting the development of hypocretin/orexin receptor 1 (OX1) antagonists for these indications. Experiments using the OX1 antagonist SB-334867 and mutant mice have involved the OX1 receptor in mediating the compulsive reinstatement of drug seeking for ethanol, nicotine, cocaine, cannabinoids and morphine. More recently, data have been generated with the novel selective OX1 antagonists GSK1059865 and ACT-335827 on behavioral and cardiovascular response to stressors and panic-inducing agents in animals. Concluding, while waiting for pharmacologic data to become available in humans, risks and benefits for the development of an OX1 receptor antagonist for Binge Eating and Anxiety Disorders are discussed.

## Introduction

Hypocretin/orexin is an hypothalamic neuropeptide consisting of two forms, A and B, containing 33 and 28 amino acids respectively, and binding to two G-protein coupled receptors, OX1 (or hcrt-1) and OX2 (or hcrt-2) (de Lecea et al., 1998; Sakurai et al., 1998).

The hypocretin/orexin peptide is produced in a small population of neurons located in the dorsomedial—perifornical hypothalamic area (DMH/PeF) and in the lateral hypothalamic nucleus (LH). Positive nerve fibers and terminals can be found in several areas of the central and peripheral nervous system, including vagus nerve, spinal cord, brainstem, hypothalamus, thalamus, limbic system, and some cortical regions (Peyron et al., 1998; Heinonen et al., 2008). OX/hcrt receptors show a similarly extended distribution (Marcus et al., 2001), suggesting a potential relevant role of hypocretins/orexins as regulatory peptides for several adaptive and limbic system-controlled functions (Johnson et al., 2012a,b; Mahler et al., 2012; Boutrel et al., 2013; Tsujino and Sakurai, 2013).

The OX1 receptor contains 425 amino acids while the OX2 receptor contains 444 amino acids with a sequence similarity between the two receptors of 68% (Sakurai et al., 1998). While both receptors are coupled with Gq-protein, only the OX1 receptor is additionally coupled with Gs-protein. Receptor activation increases intracellular calcium levels via a Gq –dependent phospholipase C (PLC)-mediated increase of inositol-1,4,5-triphosphate (Lund et al., 2000) and diacylglycerol, resulting in the activation of the δ form of the protein kinase C, eventually engaging the ERK phosphorylation pathway (Ekholm et al., 2007).

OX1 and OX2 receptors are differentially distributed throughout the mammalian brain (Marcus et al., 2001), matching the distribution of hypocretin/orexin terminals. Evidence for a functional segregation of the two receptors was recently obtained using pharmacological magnetic resonance imaging (phMRI) in rats. The activating effects of amphetamine were differentially attenuated by pre-treatment with the selective OX2 receptor antagonist JNJ-1037049 or with the novel OX1 receptor antagonist GSK1059865: JNJ-1037049 attenuated the amphetamine effects in frontal cortex and thalamus, areas involved in arousal, while GSK1059865 reduced the activation in the extended amygdala, BNST and ventral striatum; all brain areas involved in stress and motivation (Gozzi et al., 2011). These differences in functional maps, together with differences in behavioral profiles support a rationale for exploring different indications for novel therapeutics that selectively target either OX1 or OX2 receptors. However, while the role of the OX2 receptor in sleep and arousal is strongly supported by the available experimental evidence (Gatfield et al., 2010), the therapeutic potential of selective antagonism of the OX1 receptor is still under evaluation (Gotter et al., 2012).

The better understanding of the biology of the hypocretin/orexin system has promoted drug discovery programs in several pharmaceutical companies, resulting in a series of patents and compounds with different selectivity and *in vitro* characteristics (Faedo et al., 2012; Lebold et al., 2013). As shown above, some compounds were used as pharmacologic tools to explore OX1- and OX2-dependent neurotransmission *in vivo*. Few compounds were successfully progressed in humans, in particular the dual OX1-OX2 receptor antagonist (DORA) almorexant (Hoever et al., 2012), SB-649868 (Bettica et al., 2012), and suvorexant (Herring et al., 2012). Only suvorexant went successfully through Phase 3 development and it was filed in USA as new treatment for insomnia in 2013.

The first pharmacological tool used as OX1 receptor antagonist was SB-334867 (Jones et al., 2001; Smart et al., 2001). Recently, other compounds have been proposed: GSK1059865 (Alvaro et al., 2009; Gozzi et al., 2011), 2,5 di-substituted piperidines (Jiang et al., 2012) and ACT-335827 (Steiner et al., 2013).

In this review we address the evidence, mostly collected with pharmacologic tools, for a preferential role of the OX1-mediated neurotransmission in compulsive behavior, particularly in relation to addiction and binge eating, and in anxiety.

## Hypocretin/orexin and the OX1 receptor in drug addiction-like and compulsive eating behaviors

Several preclinical findings indicated the involvement of the hypocretin/orexin system in compulsive and repetitive behavior as well as in the control of goal-oriented behavior. Recent excellent reviews summaries the evidence collected in more than hundred articles indicting that the hypocretin/orexin system in the lateral hypothalamus (Harris et al., 2005) is involved in the behavioral addiction-like dysregulations associated with exposure to cocaine, amphetamine, morphine, heroin, nicotine, ethanol and cannabinoids in rodents (Espana et al., 2011; Mahler et al., 2012; Boutrel et al., 2013; Flores et al., 2013), as well as in the excessive intake of palatable food associated with binge eating (Tsujino and Sakurai, 2013).

Data supporting the hypocretin/orexin involvement in the effects of addictive drugs was initially obtained in mice carrying a null mutation (KO) of the hypocretin/orexin peptide, showing reduced signs of withdrawal from morphine (Georgescu et al., 2003). Subsequently, impaired conditioned place preference for morphine (Narita et al., 2006) and for nicotine (Plaza-Zabala et al., 2012) was demonstrated in rodents. More recently, studies in KO mice with deletion of the OX1 receptor showed reduced cocaine and cannabinoid self-administration and the blockade of reinstatement of drug taking after abstinence (Hollander et al., 2012; Flores et al., 2013), indicating a critical role for OX1 receptors in mediating reinstatement of drug seeking.

In rodents SB-334867, a preferential OX1 receptor antagonist, reduced sensitization, drug seeking behavior and withdrawal syndrome in rodents exposed to ethanol, nicotine, morphine, and cocaine. These and other findings were extensively described in recent reviews (Mahler et al., 2012; Boutrel et al., 2013). Of particular interest is the fact that SB-334867 consistently attenuated the compulsive behavior associated with the reinstatement of drug seeking, induced by either acute stress or cues associated previously with drug taking, a phenomenon observed for ethanol, nicotine, cocaine, cannabinoids and morphine.

Recently, the highly selective OX1 receptor antagonist GSK1059865 (5-bromo-N-[(2S,5S)-1-(3-fluoro-2-methoxybenzoyl)-5-methylpiperidin-2-yl]methyl-pyridin-2-amine) was characterized within the GSK collection (Alvaro et al., 2009). GSK1059865 at the dose of 25 mg/kg i.p. (estimated to fully occupy the OX1 receptors in the brain of the rat) only marginally modified the physiological sleep of rats, indicating a weak hypnotic effect (Gozzi et al., 2011; Piccoli et al., 2012) and confirming difference vs. OX2 receptor blockade (Mieda et al., 2011). Conversely, at 10 and 30 mg/kg i.p. doses, GSK1059865 significantly antagonized the cocaine effect in a conditioned place-preference paradigm (Gozzi et al., 2011). These results are in line with the proposed role of selective OX1 receptor antagonism in preventing relapse to drug seeking but not inducing sleep.

OX1 receptors were also recently involved in mediating the binge episodes of compulsive eating (Avena and Bocarsly, 2012), also defined as “food addiction,” another compulsive behavior increasingly common among obese individuals (Volkow and Wise, 2005; Pedram et al., 2013). Although it was initially shown that the acute central administration of orexin-A stimulates feeding behavior by acting on specific hypothalamic circuits (Friederich et al., 2013), hypocretin/orexin-induced food intake appears to be influenced by several other factors, including the palatability of the food, the energy balance, the level of arousal and the emotional status (Yamanaka et al., 2003; Zheng et al., 2007; Choi et al., 2010; Tsujino and Sakurai, 2013). This suggests that the hypocretin/orexin system can activate more complex behavioral patterns than the sole increase of food intake (Mahler et al., 2012). Indeed, recent studies indicate a possible involvement of hypocretin/orexin dysregulation in compulsive intake of palatable food (Smith and Robbins, 2013).

Compulsive eating can be elicited in rodents by alternating periods of regular access to food with periods of food restriction over a few weeks, a specific chronic stressful condition that can produce binge episodes when a large amount of palatable food becomes suddenly available. The model was pharmacologically validated in rats, showing an inhibitory effect of topiramate on the compulsive food intake (Cifani et al., 2009) similar to that observed in human binge eaters (McElroy et al., 2003). Although data regarding the involvement of the hypocretin/orexin pathway in this experimental procedure of binge eating were not available, we studied the effect of GSK1059865 as a tool to assess the relevance of the OX1 receptors (Piccoli et al., 2012). Interestingly, GSK1059865, at the doses of 10 and 30 mg/kg, was not able to inhibit highly palatable food intake in control animals (not exposed to cyclic food restriction), confirming the minor effect of OX1 receptor blockade on natural reward when it occurs under physiological conditions. On the contrary, GSK1059865 potently inhibited the compulsive eating behavior in rats exposed to chronic stress/food restriction (Piccoli et al., 2012). Interestingly, regular food intake was also inhibited by the OX1 antagonist SB-334867 in rats genetically prone to obesity but not in control rats (White et al., 2005). These findings confirmed the role of the OX1 receptor-mediated transmission in attenuating the excessive drive produced by craving associated to distress that was also observed with addictive drugs.

Interestingly binge eating was also inhibited by the DORA SB-649868, but not by the selective OX2 receptor antagonist JNJ-10397049, suggesting that the effects of SB-649868 are probably due to the OX1 component of its mechanism of action (Piccoli et al., 2012). Intriguingly, the lack of effect of almorexant in animals exposed only to acute stress suggested that the procedure of alternating periods of food restriction is critical for the engagement of the hypocretin/orexin system in promoting compulsive eating of highly palatable food (Funabashi et al., 2009; Pankevich et al., 2010). This observation suggests that in this paradigm DORA and OX1 antagonists do not work primarily via an anti-stress effect. This is not surprising, given the complex role of hypocretin/orexin in the maintenance of energy balance and the sensitivity of hypocretin/orexin neurons to directly respond to the changes of circulating glucose levels and to endocrine signals (Tsujino and Sakurai, 2013).

Overall, the results obtained with GSK1059865 confirm that selective OX1 receptor antagonism is not directly affecting the reward pathways involved in hedonic eating, but rather support a role in the compulsive aspect of food intake, those that probably account for the development and persistence of abnormal eating behaviors in binge eaters and, possibly, in bulimic patients. In addition, these data highlight the need to re-evaluate the putative OX1 receptor antagonism profile, so far based mostly on SB-334867 (Haynes et al., 2000), a compound whose selectivity at high dose and stability have been under discussion (Hollander et al., 2012; McElhinny et al., 2012).

To date a limited number of human biomarker studies support a role for the hypocretin/orexin system in the behavioral dysregulation that characterizes addiction, and none of them used pharmacologic agents. Changes of hypocretin/orexin levels in blood were observed in alcoholics during alcohol withdrawal, showing positive association with distress scores (von der Goltz et al., 2011), while negative association was observed with craving scores in abstinent smokers (von der Goltze et al., 2010). Increased expression of hypocretin/orexin levels was also found in the in peripheral blood of cigarette smokers and cannabis abusers (Rotter et al., 2012). While the interpretation of these finding is still unclear, subjects affected by narcolepsy were studied for their liability to addiction with the hope to get more informative results. Accordingly, narcolepsy is commonly associated with mutations in the hypocretin/orexin gene (Peyron et al., 1998), resulting in a constitutive deficiency of the peptide, similar to that obtained in hypocretin/orexin KO mice. Subtle differences in reward processing and risk-taking behavior were reported in narcoleptic subjects, but the prevalence of tobacco smoking in these patients was not different from that of the normal population (Bayard and Dauvilliers, 2013). There results can be seen at variance with evidence of reduced effects of addictive drugs in hypocretin/orexin KO mice (for review see Mahler et al., 2012; Boutrel et al., 2013). Interestingly, narcoleptic subjects declared using cigarette smoking and nicotine patches as self-medication to reduce sleepiness and increase arousal (Ebben and Krieger, 2012). Altogether, the findings indicate complexity in the relationship between addictive behaviors and dysfunctional hypocretin/orexin system in humans, supporting the need of additional, more focused translational studies.

## Hypocretin/orexin and the OX1 receptor in anxiety

Textbooks of physiology describe the posterior and perifornical regions of the hypothalamus as the part of the limbic circuit that controls “fight-or-flight” reactions in response to an imminent threat (Hess and Akert, 1955). As mentioned above, neurons that produce hypocretin/oirexin are located in the perifornical region (Peyron et al., 1998) and project to the majority of the limbic brain structures involved in the fear, stress and anxiety circuit (Shin and Liberzon, 2009), suggesting a possible role of hypocretin/orexin in controlling not only wakefulness and arousal, but also fear, anxiety, and stress responses (Johnson et al., 2012a; Sears et al., 2013).

This hypothesis was explored over the years and summarized in recent articles and reviews (Bisetti et al., 2006; Mathew et al., 2008; Johnson et al., 2010, 2012a). In these contributions interested readers can find the evidence supporting the role of hypocretin/orexin neurons in orchestrating the autonomic, respiratory, cardiovascular and behavioral responses to stressful and panic-inducing stimuli.

The working hypothesis implies that stressful stimuli (or high endogenous levels of anxiogenic mediators) would increase the activity on hypocretin/orexin neurons, which in turn will release more hypocretin/orexin in their terminal fields located in brain limbic regions that regulate emotion and stress response. Then hypocretin/orexin will be shifting the level of activation of the fear, stress and anxiety circuit toward a higher level of arousal, which includes vegetative, endocrine and behavioral phenomena typical of anxiety and panic states. An abnormal persistence of excessive release of hypocretin/orexin could be seen as a critical factor in the maintenance of high arousal and anxiety, as well as a liability to relapse into panic episodes in predisposed individuals, suggesting a potential critical pathophysiological role for anxiety.

Data in humans showed an increased release of extracellular hypocretin/orexin driven by emotional stimuli in the amygdala of subjects suffering from treatment-resistant temporal lobe epilepsy that were implanted with microdialysis probes (Blouin et al., 2013). In this study the levels of hypocretin/orexin increased during wake and decreased during sleep, but the highest peaks were observed during acute emotional activations of both positive and negative valence. The amygdala is considered a key structure for processing salience and negative emotion, which is associated with pathologic anxiety. In rodents microinjection of hypocretin/orexin into the amygdala increases anxiety-like behavior (Avolio et al., 2011). Interestingly, abnormal high levels of hypocretin/orexin were found in the cerebro-spinal fluid (CSF) of patients with Panic Anxiety Disorders, suggesting a possible hyperactivity state (Johnson et al., 2010). In another study increased levels of hypocretin/orexin were measured in the peripheral blood of subjects with chronic obstructive pulmonary disorder (COPD), a condition associated with hypercapnia, acidosis and a 10-fold increased risk of panic attacks (Zhu et al., 2011).

Converging findings suggest that the anxiogenic properties of hypocretin/orexin are primarily mediated by the engagement of OX1 receptors. In rodents the autonomic and behavioral responses to stress were attenuated by pre-treatments with OX1 receptor antagonists, such as SB-334867 (Johnson et al., 2010, 2012b), GSK 1034865 (Gozzi et al., 2011) and ACT-335827 (Steiner et al., 2013), or with a DORA, such as almorexant (Steiner et al., 2012). Significant attenuation of anxiety-like responses was observed in paradigms including fear conditioning (Sears et al., 2013; Steiner et al., 2013), panicogenic lactate infusion (Johnson et al., 2010), hypercapnia (Li et al., 2010; Johnson et al., 2012b), administration of FG-7142 (Johnson et al., 2012a), high dose nicotine (Plaza-Zabala et al., 2010), and yohimbine (Richards et al., 2008). Recent evidence using OX1 and OX2 KO mice showed a critical role of OX1 receptors located in the locus coeruleus in mediating cued fear-conditioning learning and threat memory formation (Sears et al., 2013; Soya et al., 2013).

Evidence that hypocretin/orexin-containing neurons receive inputs from other neurons producing the anxiogenic peptide corticotropin releasing factor (CRF) and that hypocretin/orexin neurons projects to CRF-rich brain regions suggested the possibility that these two peptidergic systems are functionally entangled in the control of the stress response (Ida et al., 2000; Pañeda et al., 2005). However, if this hypothesis is correct, the anxiolytic properties of CRF-1 antagonists (Zorrilla and Koob, 2004) and those of OX1 antagonists would overlap, showing similar profiles. Interestingly, a recent study using phMRI in rats suggests that the involvement of CRF-1 and OX1 in stress responses can be functionally segregated. In this experiment a phMRI activation brain map was elicited in rats with yohimbine at doses known to produce anxiogenic effects. Pre-treatments with either CP-154,526, a selective CRF-1 antagonist (Seymour et al., 2003), or the selective OX1 receptor antagonist GSK1059865 (Gozzi et al., 2011) were performed. The yohimbine activation brain map was attenuated by CP-154,526 in the motor, cingulate, retrosplenial, dorsal prefrontal cortex, dorsal portions of the caudate-putamen, and in the amygdala. Differently, GSK1059865 attenuated the yohimbine activation map in the nucleus accumbens, septum, dorsal thalamus, amygdala, ventral hippocampus, orbitofrontal, prefrontal, insular, cingulate retrosplenial, and piriform cortex (Gozzi et al., 2013). Overall, the OX1 receptor antagonist exerted a more extensive effect on the fear, stress and anxiety circuit than the CRF1 antagonist, attenuating the activations of regions of the dopaminergic mesolimbic system. In line with the latter observation, a dissociation was found between the effects of OX1 and CRF-1 antagonists on the rat mesolimbic dopamine system in stress-induced cocaine (Wang et al., 2009) and nicotine seeking (Plaza-Zabala et al., 2012).

Interestingly, in some studies both OX1 antagonists and DORAs did not show anxiolytic effects in specific behavioral tests (e.g., the elevated plus maze) (Steiner et al., 2012; Rodgers et al., 2013). These data are in line with the hypothesis that hypocretin/orexin receptor antagonists do not alter basal anxiety levels in rodents, but exert anxiolytic-like properties when anxiety levels are transiently exacerbated by potent stimuli such as acidosis/hypercapnia.

## Limitations and conclusion

The rationale for considering OX1 receptors as a possible target in conditions such as Anxiety Disorders, Drug Addiction, and Binge Eating was reviewed. Based on the current knowledge on mechanism of action, OX1 receptor antagonists should have fewer development risks than DORAs. As highlighted by Scammell and Winrow (2011) and Boutrel et al. (2013) for DORAs, the chronic simultaneous blockade of both hypocretin/orexin receptors can potentially: (1) induce narcoleptic-like symptoms, including catalepsy; (2) impair goal oriented decision-making; (3) reduce pleasure associated with rewarding activities; (4) induce sedation, sleepiness and impaired coping responses under acute emergency or stress; (5) impact on basal metabolism with increased body weight. However, so far the human experience with the DORAs suvorexant, SB-649868 and almorexant is very encouraging, showing minimal occurrence of the above mentioned adverse events and, in particular, no induction of narcoleptic eoisodes or impairment of decision making performance when tested within the dose range currently proposed. However, more data on larger populations and higher doses are required to reach a final conclusion.

Interestingly, treatments with selective OX1 antagonists are not expected to share the same risks of DORAs since only the OX2 receptor is primarily associated with narcolepsy which was demonstrated for dogs carrying a disruptive genetic mutation of this receptor (Wu et al., 2011). The additional potential benefits of an OX1 antagonist include: (1) A preclinical anxiolytic profile that differentiates it from benzodiazepines, serotonin-uptake inhibitors, and CRF-1 antagonists; (2) The capacity of reducing hyper-arousal states associated to acute and severe anxiety episodes with relevant physical symptoms, such as panic attacks or withdrawal from addictive drugs; (3) The attenuation of compulsive and repetitive behaviors associated with sensitization, stress-relieve, or drug seeking; (4) The lack of effects on desire of natural rewards, and (5) the lack of sleep-inducing effects.

These considerations are based on a limited dataset of preclinical studies, only recently conducted with the new generation of selective compounds. Chronic dosing studies are missing and therefore information of long term risks and benefits are not available yet. In addition, no selective OX1 receptor antagonists have been tested in humans so far and the translational barrier is still unclear. However, the promising therapeutic potential in the modulation of anxiety and compulsive behaviors is stimulating further basic research and is encouraging active investments of pharmaceutical companies.

### Conflict of interest statement

Emilio Merlo Pich is full time employee of F. Hoffman-La Roche. The other author declares that the research was conducted in the absence of any commercial or financial relationships that could be construed as a potential conflict of interest.

## References

[B1] Alvaro G. Amantini D. Stasi L. (2009). Pyridine Derivatives Used to Treat Orexin Related Disorders. WO 2009124956. PCT Int. Appl.

[B2] Avena N. M. Bocarsly M. E. (2012). Dysregulation of brain reward systems in eating disorders: neurochemical information from animal models of binge eating, bulimia nervosa, and anorexia nervosa. Neuropharmacology 63, 87–96 10.1016/j.neuropharm.2011.11.010 22138162 PMC3366171

[B3] Avolio E. Alò R. Carelli A. Canonaco M. (2011). Amygdalar orexinergic-GABAergic interactions regulate anxiety behaviors of the Syrian golden hamster. Behav. Brain Res. 218, 288–295 10.1016/j.bbr.2010.11.014 21074570

[B4] Bayard S. Dauvilliers Y. A. (2013). Reward-based behaviors and emotional processing in human with narcolepsy-cataplexy. Front. Behav. Neurosci. 7:50 10.3389/fnbeh.2013.00050 23734110 PMC3661950

[B5] Bettica P. Squassante L. Zamuner S. Nucci G. Danker-Hopfe D. Ratti E. (2012). The orexin antagonist SB-649868 promotes and maintain sleep in men with primary insomnia. Sleep 35, 1097–1104 10.5665/sleep.1996 22851805 PMC3397789

[B6] Bisetti A. Cvetkovic V. Serafin M. Bayer L. Machard D. Jones B. E. (2006). Excitatory action of hypocretin/orexin on neurons of the central medial amygdala. Neuroscience 142, 999–1004 10.1016/j.neuroscience.2006.07.018 16996221

[B7] Blouin A. M. Fried I. Wilson C. L. Staba R. J. Behnke E. J. Lam H. A. (2013). Human hypocretin and melanin-concentrating hormone levels are linked to emotion and social interaction. Nat. Commun. 4, 1547–1553 10.1038/ncomms2461 23462990 PMC3595130

[B8] Boutrel B. Steiner N. Halfon O. (2013). The hypocretins and the reward function: what have we learned so far? Front. Behav. Neurosci. 7:59 10.3389/fnbeh.2013.00059 23781178 PMC3680710

[B9] Choi D. L. Davis J. F. Fitzgerald M. E. Benoit S. C. (2010). The role of orexin-A in food motivation, reward-based feeding behavior and food-induced neuronal activation in rats. Neuroscience 167, 11–20 10.1016/j.neuroscience.2010.02.002 20149847

[B10] Cifani C. Polidori C. Melotto S. Ciccocioppo R. Massi M. (2009). A preclinical model of binge eating elicited by yo-yo dieting and stressful exposure to food: effect of sibutramine, fluoxetine, topiramate, and midazolam. Psychopharmacology (Berl.) 204, 113–125 10.1007/s00213-008-1442-y 19125237

[B11] de Lecea L. Kilduff T. S. Peyron C. Gao X. B. Foye P. E. Danielson P. E. (1998). The hypocretins: hypothalamus-specific peptides with neuroexcitatory activity. Proc. Natl. Acad. Sci. U.S.A. 95, 322–327 10.1073/pnas.95.1.322 9419374 PMC18213

[B12] Ebben M. R. Krieger A. C. (2012). Narcolepsy with cataplexy masked by the use of nicotine. J. Clin. Sleep Med. 8, 195–196 10.5664/jcsm.1780 22505866 PMC3311418

[B13] Ekholm M. E. Johannson L. Kukkonen J. P. (2007). IP3 independent signalling of OX1 orexin/hypocretin receptor to Ca2+ influx and ERK. Biochem. Biophys. Res. Commun. 353, 475–480 10.1016/j.bbrc.2006.12.045 17188243

[B14] Espana R. A. Melchior J. R. Roberts D. C. Jones S. R. (2011). Hypocretin 1/orexin A in the ventral tegmental area enhances dopamine responses to cocaine and promotes cocaine self-administration. Psychopharmacology (Berl.) 214, 415–426 10.1007/s00213-010-2048-8 20959967 PMC3085140

[B15] Faedo S. Perdonà E. Antolini M. di Fabio R. Merlo Pich E. Corsi M. (2012). Functional and binding kinetic studies make a distinction between OX 1 and OX2 orexin receptor antagonists. Eur. J. Pharmacol. 692, 1–9 10.1016/j.ejphar.2012.07.007 22796453

[B16] Flores A. Maldonado R. Berrendero F. (2013). The hypocretin/orexin receptor-1 as a novel target to modulate cannabinoid reward. Biol. Psychiatry 3223, 590–598 10.1016/j.biopsych.2013.06.012 23896204

[B17] Friederich H. C. Wu M. Simon J. J. Herzog W. (2013). Neurocircuit function in eating disorders. Int. J. Eat. Disord. 46, 425–432 10.1002/eat.22099 23658085

[B18] Funabashi T. Hagiwara H. Mogi K. Mitsushima D. Shinohara K. Kimura F. (2009). Sex differences in the responses of orexin neurons in the lateral hypothalamic area and feeding behavior to fasting. Neurosci. Lett. 463, 31–34 10.1016/j.neulet.2009.07.035 19616070

[B19] Gatfield J. Brisbare-Roch C. Jenck F. Boss C. (2010). Orexin receptor antagonists: a new concept in CNS disorders? ChemMedChem 5, 1197–1214 10.1002/cmdc.201000132 20544785

[B20] Georgescu D. Zachariou V. Barrot M. Mieda M. Willie J. T. Eisch A. J. (2003). Involvement of the lateral hypothalamic peptide orexin in morphine dependence and with- drawal. J. Neurosci. 23, 3106–3111 10.1002/micr.10128 12716916 PMC6742290

[B21] Gotter A. L. Roecker A. J. Hargreaves R. Coleman P. J. Winrow C. J. Renger J. J. (2012). Orexin receptors as therapeutic drug targets. Prog. Brain Res. 198, 163–188 10.1016/B978-0-444-59489-1.00010-0 22813974

[B22] Gozzi A. Lepore S. Vicentini E. Merlo-Pich E. Bifone A. (2013). Differential effect of orexin-1 and CRF-1 antagonism on stress circuits: a fMRI study in the rat with the pharmacological stressor yohimbine. Neuropsychopharmacology 38, 2120–2130 10.1038/npp.2013.109 23736277 PMC3773661

[B23] Gozzi A. Turrini G. Piccoli L. Massagrande M. Amantini D. Antolini M. (2011). Functional magnetic resonance imaging reveals different neural substrates for the effects of orexin-1 and orexin-2 receptor antagonists. PLoS ONE 6:e16406 10.1371/journal.pone.0016406 21307957 PMC3030585

[B24] Harris G. C. Wimmer M. Aston-Jones G. (2005). A role for lateral hypothalamic orexin neurons in reward seeking. Nature 437, 556–559 10.1038/nature04071 16100511

[B25] Haynes A. C. Jackson B. Chapman H. Tadayyon M. Johns A. Porter R. A. (2000). A selective orexin-1 receptor antagonist reduces food consumption in male and female rats. Regul. Pept. 96, 45–51 10.1016/S0167-0115(00)00199-3 11102651

[B26] Heinonen M. V. Purhonon A. K. Makela K. A. Herzig K. H. (2008). Functions of orexin in peripheral tissue. Acta Physiol. (Oxf.) 192, 471–485 10.1111/j.1748-1716.2008.01836.x 18294339

[B27] Herring W. J. Snyder E. Budd K. Hutzelmann J. Snavely D. Liu K. (2012). Orexin receptor antagonism for treatment of insomnia: a randomized clinical trial of suvorexant. Neurology 79, 2265–2274 10.1212/WNL.0b013e31827688ee 23197752

[B28] Hess W. R. Akert K. (1955). Experimental data on role of hypothalamus in mechanism of emotional behavior. AMA Arch. Neurol. Psychiatry 73, 127–129 10.1001/archneurpsyc.1955.02330080005003 13227662

[B29] Hoever P. Dorffner G. Bene H. Penzel T. Danker-Hopfe H. Barbanoj M. J. (2012). Orexin receptor antagonism, a new sleep-enabling paradigm: a proof-of-concept clinical trial. Clin. Pharmacol. Ther. 91, 975–985 10.1038/clpt.2011.370 22549286 PMC3370822

[B31] Hollander J. A. Pham D. Fowler C. D. Kenny P. J. (2012). Hypocretin-1 receptors regulate the reinforcing and reward-enhancing effects of cocaine: pharmacological and behavioral genetics evidence. Front. Behav. Neurosci. 6:47 10.3389/fnbeh.2012.00047 22837742 PMC3402880

[B32] Ida T. Nakahara K. Murakami T. Hanada R. Nakazato M. Murakami N. (2000). Possible involvement of orexin in the stress reaction in rats. Biochem. Biophys. Res. Commun. 270, 318–323 10.1006/bbrc.2000.2412 10733946

[B33] Jiang R. Song X. Bali P. Smith A. Bayona C. R. Lin L. (2012). Disubstituted piperidines as potent orexin (hypocretin) receptor antagonists. Bioorg. Med. Chem. Lett. 12, 3890–3894 10.1016/j.bmcl.2012.04.122 22617492 PMC3383661

[B34] Johnson P. L. Molosh A. Fitz S. D. Truitt W. A. Shekhar A. (2012a). Orexin, stress, and anxiety/panic states. Prog. Brain Res. 198, 133–161 10.1016/B978-0-444-59489-1.00009-4 22813973 PMC3665356

[B35] Johnson P. L. Samuels B. C. Fitz S. D. Federici L. M. Hammes N. Early M. C. (2012b). Orexin 1 receptors are a novel target to modulate panic responses and the panic brain network. Physiol. Behav. 107, 733–742 10.1016/j.physbeh.2012.04.016 22554617 PMC3472124

[B36] Johnson P. L. Truitt W. Fitz S. D. Minick P. E. Dietrich A. Sanghani S. (2010). A key role for orexin in panic anxiety. Nat. Med. 16, 111–115 10.1038/nm.2075 20037593 PMC2832844

[B37] Jones D. N. C. Gartlon J. Parker F. Taylor S. G. Routledge C. Hemmati P. (2001). Effects of centrally administered orexin-B and orexin-A: a role for orexin-1 receptors in orexin-B-induced hyperactivity. Psychopharmacology 153, 210–218 10.1007/s002130000551 11205421

[B38] Lebold T. P. Bonaventure P. Shireman B. T. (2013). Selective orexin receptor antagonists. Bioorg. Med. Chem. Lett. 223, 4761–4769 10.1016/j.bmcl.2013.06.057 23891187

[B39] Li Y. Li S. Wei C. Wang H. Sui N. Kirouac G. J. (2010). Orexins in the paraventricular nucleus of the thalamus mediate anxiety-like responses in rats. Psychopharmacology 212, 251–265 10.1007/s00213-010-1948-y 20645079

[B40] Lund P. E. Sariatmadari R. Uustare A. Detheux M. Parmentier M. Kukkonen J. P. (2000). The orexin OX1 receptor activates a novel CA2+ influx pathway necessary for coupling to phospholypase C. J. Biol. Chem. 275, 30806–30812 10.1074/jbc.M002603200 10880509

[B41] Mahler S. V. Smith R. J. Moorman D. E. Sartor G. C. Aston-Jones G. (2012). Multiple roles for orexin/hypocretin in addiction. Prog. Brain Res. 198, 79–121 10.1016/B978-0-444-59489-1.00007-0 22813971 PMC3643893

[B42] Marcus J. N. Aschkenasi C. J. Lee C. E. Chemelli R. M. Saper C. B. Yanagisawa M. (2001). Differential expression of orexin receptors 1 and 2 in the rat brain. J. Comp. Neurol. 435, 6–25 10.1002/cne.1190 11370008

[B43] Mathew S. J. Price R. B. Charney D. S. (2008). Recent advances in the neurobiology of anxiety disorders: implications for novel therapeutics. Am. J. Med. Genet. C Semin. Med. Genet. 148C, 89–98 10.1002/ajmg.c.30172 18412102

[B44] McElhinny C. J. Jr. Lewin A. H. Mascarella S. W. Runyon S. Brieaddy L. Carroll F. I. (2012). Hydrolytic instability of the important orexin 1 receptor antagonist SB-334867: possible confounding effects on *in vivo* and *in vitro* studies. Bioorg. Med. Chem. Lett. 22, 6661–6664 10.1016/j.bmcl.2012.08.109 23031594

[B45] McElroy S. L. Arnold L. M. Shapira N. A. Keck P. E. Jr. Rosenthal N. R. Karim M. R. (2003). Topiramate in the treatment of binge eating disorder associated with obesity: a randomized, placebo-controlled trial. Am. J. Psychiatry 160, 255–261 10.1176/appi.ajp.160.2.255 12562571

[B46] Mieda M. Hasegawa E. Kisanuki Y. Y. Sinton C. M. Yanagisawa M. Sakurai T. (2011). Differential roles of orexin receptor-1 and -2 in the regulation of non-REM and REM sleep. J. Neurosci. 31, 6518–6526 10.1523/JNEUROSCI.6506-10.2011 21525292 PMC3732784

[B47] Narita M. Nagumo Y. Hashimoto S. Khotib J. Miyatake M. Sakurai T. (2006). Direct involvement of orexinergic systems in the activation of the mesolimbic dopamine pathway and related behaviours induced by morphine. J. Neurosci. 26, 398–405 10.1523/JNEUROSCI.2761-05.2006 16407535 PMC6674410

[B48] Pañeda C. Winsky-Sommerer R. Boutrel B. de Lecea L. (2005). The corticotropin-releasing factor-hypocretin connection: implications in stress response and addiction. Drug News Perspect. 18, 250–255 10.1358/dnp.2005.18.4.908659 16034481

[B49] Pankevich D. E. Teegarden S. L. Hedin A. D. Jensen C. L. Bale T. L. (2010). Caloric restriction experience reprograms stress and orexigenic pathways and promotes binge eating. J. Neurosci. 30, 16399–16407 10.1523/JNEUROSCI.1955-10.2010 21123586 PMC3034235

[B50] Pedram P. Wadden D. Amini P. Gulliver W. Randell E. Cahill F. (2013). Food addiction: its prevalence and significant association with obesity in the general population. PLoS ONE 8:e74832 10.1371/journal.pone.0074832 24023964 PMC3762779

[B51] Peyron C. Tighe D. K. van den Pol A. N. de Lecea L. Heller H. C. Sutcliffe J. G. (1998). Neurons containing hypocretin (orexin) project to multiple neuronal systems. J. Neurosci. 18, 9996–10015 9822755 10.1523/JNEUROSCI.18-23-09996.1998 PMC6793310

[B52] Piccoli L. Micioni Di Bonaventura M. V. Cifani C. Costantini V. J. Massagrande M. Montanari D. (2012). Role of orexin-1 receptor mechanisms on compulsive food consumption in a model of binge eating in female rats. Neuropsychopharmacology 37, 1999–2011 10.1038/npp.2012.48 22569505 PMC3398727

[B53] Plaza-Zabala A. Flores A. Maldonado R. Berrendero F. (2012). Hypocretin/Orexin signaling in the hypothalamic paraventricular nucleus is essential for the expression of nicotine withdrawal. Biol. Psychiatry 71, 214–223 10.1016/j.biopsych.2011.06.025 21831361

[B54] Plaza-Zabala A. Martín-García E. de Lecea L. Maldonado R. Berrendero F. (2010). Hypocretins regulate the anxiogenic-like effects of nicotine and induce reinstatement of nicotine-seeking behavior. J. Neurosci. 30, 2300–2310 10.1523/JNEUROSCI.5724-09.2010 20147556 PMC2829191

[B55] Richards J. K. Simms J. A. Steensland P. Taha S. A. Borgland S. L. Bonci A. (2008). Inhibition of orexin-1/hypocretin-1 receptors inhibits yohimbine-induced reinstatement of ethanol and sucrose seeking in long-evans rats. Psychopharmacology (Berl.) 199, 109–117 10.1007/s00213-008-1136-5 18470506 PMC2668563

[B56] Rodgers R. J. Wright F. L. Snow N. F. Taylor L. J. (2013). Orexin-1 receptor antagonism fails to reduce anxiety-like behaviour in either plus-maze-naïve or plus-maze-experienced mice. Behav. Brain Res. 243, 213–219 10.1016/j.bbr.2012.12.064 23333844

[B57] Rotter A. Bayerlein K. Hansbauer M. Weiland J. Sperling W. Kornhuber J. (2012). Orexin A expression and promoter methylation in patients with cannabis dependence in comparison to nicotine-dependent cigarette smokers and nonsmokers. Neuropsychobiology 66, 126–133 10.1159/000339457 22846875

[B58] Sakurai T. Amemiya A. Ishii M. Matsuzaki I. Chemelli R. M. Tanaka H. (1998). Orexins and orexin receptors: a family of hypothalamic neuropeptides and G protein-coupled receptors that regulate feeding behavior. Cell 92, 573–585 10.1016/S0092-8674(00)80949-6 9491897

[B59] Scammell T. E. Winrow C. J. (2011). Orexin receptors: pharmacology and therapeutic opportunities. Annu. Rev. Pharmacol. Toxicol. 51, 243–266 10.1146/annurev-pharmtox-010510-100528 21034217 PMC3058259

[B60] Sears R. M. Fink A. E. Wigestrand M. B. Farb C. R. de Lecea L. Ledoux J. E. (2013). Orexin/hypocretin system modulates amygdala-dependent threat learning through the locus coeruleus. Proc. Natl. Acad. Sci. U.S.A. 110, 20260–20265 10.1073/pnas.1320325110 24277819 PMC3864341

[B61] Seymour P. A. Schmidt A. W. Schulz D. W. (2003). The pharmacology of CP-154,526, a non-peptide antagonist of the CRH1 receptor: a review. CNS Drug Rev. 9, 57–96 10.1111/j.1527-3458.2003.tb00244.x 12595912 PMC6741649

[B62] Shin L. M. Liberzon I. (2009). The neurocircuitry of fear, stress, and anxiety disorders. Neuropsychopharmacology 35, 169–191 10.1038/npp.2009.83 19625997 PMC3055419

[B63] Smart D. Sabido-David C. Brough S. J. Jewitt F. Johns A. Porter R. A. (2001). SB-334867-A: the first selective orexin-1 receptor antagonist. Br. J. Pharmacol. 132, 1179–1182 10.1038/sj.bjp.0703953 11250867 PMC1572677

[B64] Smith D. G. Robbins T. W. (2013). The neurobiological underpinnings of obesity and binge eating: a rationale for adopting the food addiction model. Biol. Psychiatry 73, 804–810 10.1016/j.biopsych.2012.08.026 23098895

[B65] Soya S. Shoji H. Hasegawa E. Hondo M. Miyakawa T. Yanagisawa M. (2013). Orexin receptor-1 in the locus coeruleus plays an important role in cue-dependent fear memory consolidation. J. Neurosci. 33, 14549–14557 10.1523/JNEUROSCI.1130-13.2013 24005305 PMC6618384

[B66] Steiner M. A. Gatfield J. Brisbare-Roch C. Dietrich H. Treiber A. Jenck F. (2013). Discovery and characterization of ACT-335827, an orally available, brain penetrant orexin receptor type1 selective antagonist. ChemMedChem 8, 898–903 10.1002/cmdc.201300003 23589487

[B67] Steiner M. A. Lecourt H. Jenck F. (2012). The brain orexin system and almorexant in fear-conditioned startle reactions in the rat. Psychopharmacology (Berl.) 223, 465–475 10.1007/s00213-012-2736-7 22592903

[B68] Tsujino N. Sakurai T. (2013). Role of orexin in modulating arousal, feeding, and motivation. Front. Behav. Neurosci. 7:28 10.3389/fnbeh.2013.00028 23616752 PMC3629303

[B69] Volkow N. D. Wise R. A. (2005). How can drug addiction help us understand obesity? Nat. Neurosci. 8, 555–560 10.1038/nn1452 15856062

[B70] von der Goltz C. Koopmann A. Dinter C. Richter A. Grosshans M. Fink T. (2011). Involvement of orexin in the regulation of stress, depression and reward in alcohol dependence. Horm. Behav. 60, 644–650 10.1016/j.yhbeh.2011.08.017 21945150

[B71] von der Goltz C. Koopmann A. Dinter C. Richter A. Rockenbach C. Grosshans M. (2010). Orexin and leptin are associated with nicotine craving: a link between smoking, appetite and reward. Psychoneuroendocrinology 35, 570–577 10.1016/j.psyneuen.2009.09.005 19828259

[B72] Wang B. You Z. B. Wise R. A. (2009). Reinstatement of cocaine seeking by hypocretin/orexin in the ventral tegmental area: independence from the local corticotropin-releasing factor network. Biol. Psychiatry 65, 857–862 10.1016/j.biopsych.2009.01.018 19251246 PMC2705875

[B73] White C. L. Ishii Y. Mendoza T. Upton N. Stasi L. P. Bray G. A. (2005). Effect of a selective OX1R antagonist on food intake and body weight in two strains of rats that differ in susceptibility to dietary-induced obesity. Peptides 26, 2331–2338 10.1016/j.peptides.2005.03.042 15893404

[B74] Wu M. F. Nienhuis R. Maidment N. Lam H. A. Siegel J. M. (2011). Role of the hypocretin (orexin) receptor 2 (Hcrt-r2) in the regulation of hypocretin level and cataplexy. J. Neurosci. 31, 6305–6310 10.1523/JNEUROSCI.0365-11.2011 21525270 PMC3097029

[B75] Yamanaka A. Beuckmann C. T. Willie J. T. Hara J. Tsujino N. Mieda M. (2003). Hypothalamic orexin neurons regulate arousal according to energy balance in mice. Neuron 38, 701–713 10.1016/S0896-6273(03)00331-3 12797956

[B76] Zheng H. Patterson L. M. Berthoud H. R. (2007). Orexin signaling in the ventral tegmental area is required for high-fat appetite induced by opioid stimulation of the nucleus accumbens. J. Neurosci. 27, 11075–11082 10.1523/JNEUROSCI.3542-07.2007 17928449 PMC6672863

[B77] Zhu L. Y. Summah H. Jiang H. N. Qu J. M. (2011). Plasma orexin-a levels in COPD patients with hypercapnic respiratory failure. Mediators Inflamm. 2011, 754847 10.1155/2011/754847 21765621 PMC3134321

[B78] Zorrilla E. P. Koob G. F. (2004). The therapeutic potential of CRF1 antagonists for anxiety. Expert Opin. Investig. Drugs 13, 799–828 10.1517/13543784.13.7.799 15212620

